# Relationship between the Levels of Calprotectin and Soluble Receptor for Advanced Glycation End Products with Abdominal Aortic Aneurysm Diameter: A Preliminary Clinical Trial

**DOI:** 10.3390/jcm11185448

**Published:** 2022-09-16

**Authors:** Willy Hauzer, Jan Gnus, Joanna Rosińczuk

**Affiliations:** 1 Department of Vascular Surgery, Hauzer Clinic LLC LP, 55-110 Żerniki Wroclawskie, Poland; 2 Department of Physiotherapy, Wroclaw Medical University, 55-355 Wroclaw, Poland; 3 Department of Nursing and Obstetrics, Wroclaw Medical University, 51-618 Wroclaw, Poland

**Keywords:** abdominal aortic aneurysm, human biomarkers, receptors for advanced glycation end products, calprotectin

## Abstract

An abdominal aortic aneurysm (AAA) is a dilatation of the abdominal aorta greater than 50% of the diameter of a healthy aorta. Previous experimental studies confirm the effect of calprotectin (CAL) on the onset of arterial pathology. It has been suggested that low levels of soluble receptors for advanced glycation end products (RAGEs) increase levels of cytokines that lead to the inhibition of matrix metalloproteinases (MMPs), affecting AAA formation. This study aimed to analyze the correlation of levels of RAGE and CAL with AAA diameter. A group of 32 patients aged 50–75 with diagnosed AAA was enrolled in the study. This group of patients was further divided into three subgroups based on AAA diameter: (1) <4.5 cm, (2) 4.5–5.5 cm, (3) >5.5 cm. Peripheral blood was drawn from all participants on admission to measure the serum CAL and RAGE levels. An enumeration survey was performed three months after AAA surgical treatment. CAL and RAGE plasma levels were measured with the enzyme-linked immunosorbent assay (ELISA). The median CAL levels were 2273.0 ng/mL before and 1217.0 ng/mL after treatment. There was a statistically significant decrease in CAL levels following the surgical treatment (*p* = 0.003). The correlation analysis between CAL levels and RAGE levels before and after surgical treatment showed no statistically significant correlations. In addition, there were no statistically significant correlations between CAL and RAGE levels with AAA size. In conclusion, CAL levels appear to be a significant marker in patients with AAA. There is an almost twofold decrease in CAL levels after AAA excision.

## 1. Introduction

An abdominal aortic aneurysm (AAA) is a dilatation of the abdominal aorta greater than 50% of the diameter of a healthy aorta. Factors involved in AAA formation include hypertension, smoking, collagen- and elastase-related genetic factors, atherosclerosis as an inflammatory process, and bacterial and viral factors. In addition, several biomarkers are involved in the AAA formation, describing its elastolytic and collagenolytic activities [1].

Available studies prove that calprotectin (CAL) may be a prognostic factor for cardiovascular disease [2]. CAL causes arthropathies and vascular pathologies of coronary and carotid arteries [3]. Animal experimental studies proved the importance of CAL in the pathogenesis of AAA [4]. There was also a correlation between elevated CAL levels in cardiovascular disease [5], sepsis [6], and even COVID-19 [7].

Advanced glycation end products (AGEs) and RAGEs affect pro-inflammatory mediators [8]. There are two types of RAGE, soluble RAGE and membrane-bound RAGE [9]. It has been known for many years that metalloproteinases (MMPs) are linked to the etiopathogenesis of AAA [10]. The previous studies concerning AAA confirmed the impact of AGE/RAGE-induced MMP-9 levels when macrophages were treated with RAGEs to inhibit RAGE signaling [11,12]. AGEs react with cell receptors and increase cytokine release. RAGEs bind to AGEs by reducing the production of pro-inflammatory cytokines [13].

It was suggested that low levels of RAGE increase cytokine levels that increase MMP levels and thus affect aneurysm formation. RAGE interacts with a wide range of ligands stimulating the release of pro-inflammatory cytokines involved in aneurysm formation. When binding to the AGE ligand, the soluble form of RAGE blocks RAGE, whose levels may be elevated in aneurysms [14,15,16]. This may prevent the development of vascular pathology, atherosclerosis, and aneurysms [17].

Our previous study confirmed that CAL might be a promising biomarker related to the occurrence of AAA [18]. We reported a statistically significant increase in CAL levels compared to controls without AAA. There was also a statistically significant decrease in calprotectin levels after AAA surgery. However, it mainly considered CAL levels in patients with AAA. Moreover, it was concluded that further studies are needed to confirm the role of CAL in the development and progression of the AAA unambiguously. Determination of the RAGE levels in patients before and after AAA surgery is still understudied. Additionally, comparisons of the sensitivity of CAL and RAGE biomarkers in patients with AAA and the relationship with AAA diameter are crucial. Therefore, this study aimed to compare RAGE and CAL levels before and after AAA surgery and evaluate the correlation of RAGE and CAL levels with the AAA diameter.

## 2. Materials and Methods

### 2.1. Study Design and Settings

The study involved patients of the Division of Vascular Surgery treated for AAA between October 2017 and September 2018 under the KNOW project (application no. W10/1/4/2017) funded by the Regional Specialist Hospital in Wroclaw, Research and Development Centre, Poland.

### 2.2. Ethical Considerations

The study was approved by the independent Bioethics Committee at the Research and Development Centre, Regional Specialist Hospital in Wroclaw, Poland (approval no. KB/6/2017). Each patient gave written informed consent to participate in the study. All ethical principles of the Helsinki Declaration were followed. The study was conducted following all relevant regulations and Good Clinical Practice (GCP) guidelines.

### 2.3. Study Participants

A group of 32 patients aged 50–75 with AAA diagnosis confirmed in abdominal ultrasound and/or angio-CT were included in the study group. All patients were qualified for AAA vascular surgery using the traditional open aneurysm repair (OAR). Inclusion criteria comprised: (1) AAA diagnosed using ultrasound and/or abdominal angio-CT, (2) age over 18 years, (3) Caucasian race, (4) and obtained written informed consent to participate in the study. In addition, patients who suffered from conditions requiring pharmacotherapy with immunosuppressive drugs or other medications were excluded.

The study group was divided into three subgroups according to the aneurysm’s diameter: the first subgroup consisted of patients with AAA of less than 4.5 cm in diameter, the second subgroup included patients with AAA of 4.5–5.5 cm in diameter, and the third group consisted of patients with AAA greater than 5.5 cm in diameter.

### 2.4. Research Methods

Blood was drawn on admission before surgery to measure serum levels of RAGE and CAL in all participants. Three months after the surgery, serum levels of RAGE and CAL were re-analyzed. The test material was peripheral blood collected into EDTA tubes. After centrifugation, plasma was portioned and stored at −80 °C until measurements were performed. Plasma CAL levels and plasma RAGE levels were measured using the enzyme-linked immunosorbent assay (ELISA). The measurements were performed using a SPECTROstar Nano plate reader (BMG LABTECH, Ortenberg, Germany).

### 2.5. Statistical Analysis

The statistical analysis was performed using Statistica 13 software (TIBCO, Inc., Palo Alto, California, USA). Arithmetic means, medians, standard deviations, and the range of variability (extreme values) were calculated for measurable variables. In the case of qualitative variables, their frequency (%) was calculated. All studied quantitative variables were verified with the Shapiro–Wilk test used to determine a distribution type. A comparison of the results obtained before and after the surgical intervention was performed using the nonparametric Wilcoxon test. The comparisons of results according to AAA diameter (small, medium, large) were performed using repeated measures analysis of variance (RMANOVA) by Friedman’s ranks. Spearman’s correlation analysis of selected results was also performed. A statistical significance level of α = 0.05 was used for all comparisons.

## 3. Results

The study group’s profile, including selected characteristics such as age, anthropometric data, AAA diameter, sex, chronic comorbidities, and AAA division according to size, is shown in Table 1.

The study group consisted of 32 patients (30 males, 94%) with a mean age of 70.3 years. The mean body weight was 91 kg and the BMI value of 30.5 kg/m^2^. Hypertension was present in more than 87% of participants. A much smaller percentage of patients had type 2 diabetes (14%) and heart disease (28%). Half of the patients had medium-sized aneurysms (1b) (Table 1).

**Figure 1:**
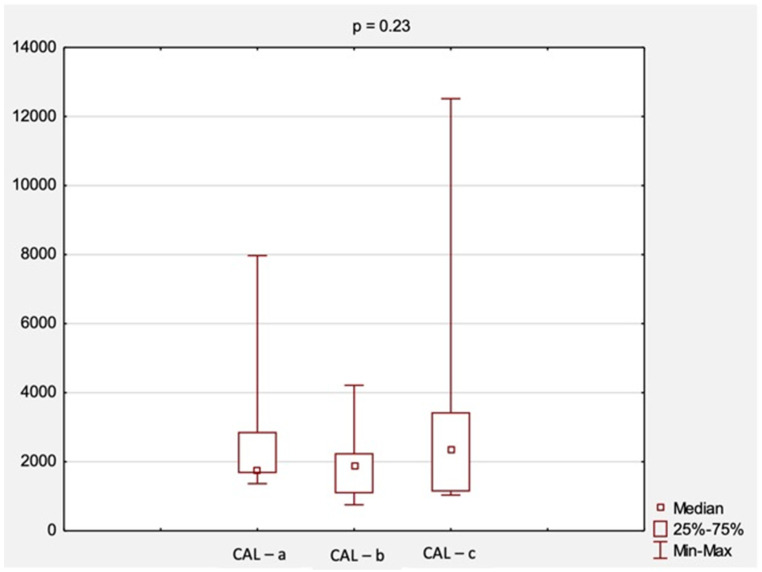
The comparison of CAL levels according to AAA diameter. *Abbreviations:* CAL, calprotectin; a, small aneurysm (<4.5 cm); b, medium aneurysm (4.5–5.5 cm); c, large aneurysm (>5.5 cm); AAA, abdominal aortic aneurysm; *p*, level of statistical significance.

A comparison of CAL levels according to aneurysm size (1a—small, 1b—medium, 1c—large) is shown in Figure 1. The highest median CAL levels were found in group 1c—2344.0 ng/mL, followed by group 1b with a value of 1865.0 ng/mL and group 1a with 1732.0 ng/mL. However, those results were not statistically significant (*p* = 0.23, Figure 1).

**Figure 2:**
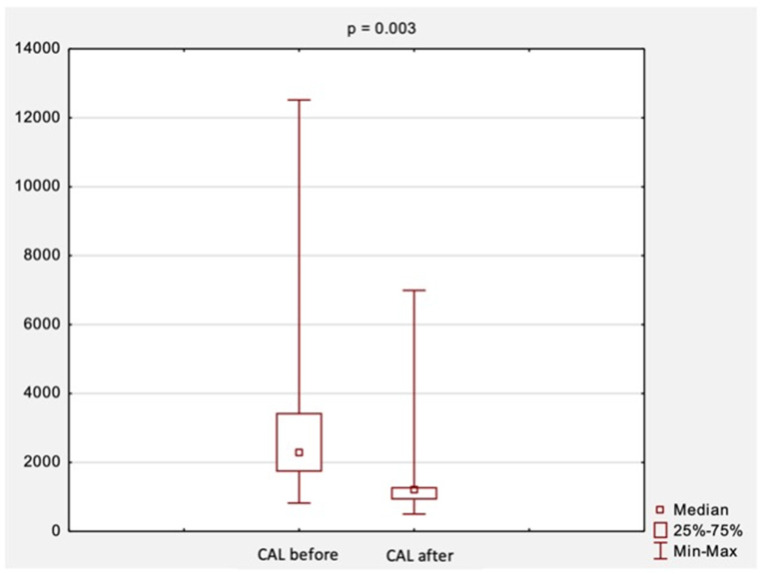
The comparison of CAL levels before and after surgical treatment. *Abbreviations:* CAL, calprotectin; *p*, level of statistical significance.

An attempt was also made to evaluate changes in CAL and RAGE levels before and after surgical treatment. A comparison of CAL levels obtained at the first and second visit is shown in Figure 2. The median CAL levels before treatment were 2273.0 ng/mL, while after treatment, they were 1217.0 ng/mL. There was a statistically significant decrease in CAL levels after surgical treatment (*p* = 0.003, Figure 2).

**Figure 3:**
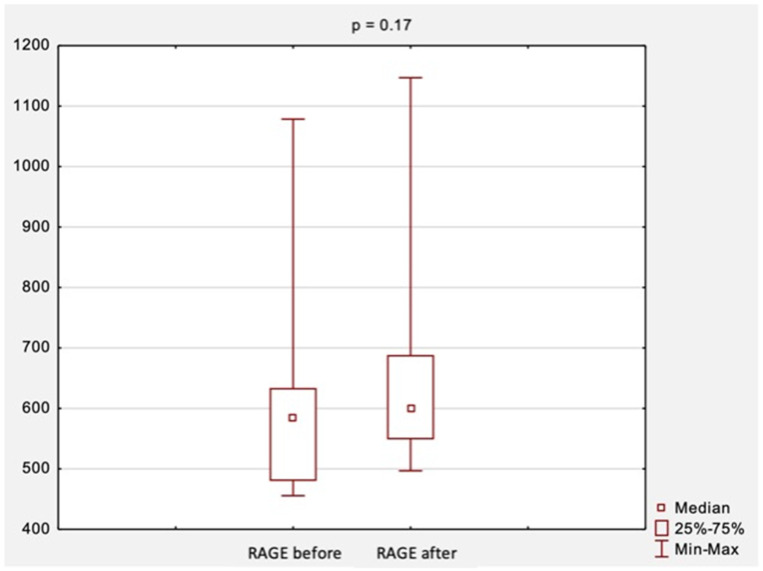
The comparison of RAGE levels before and after surgical treatment. *Abbreviations:* RAGE, receptor for advanced glycation end products; *p*, level of statistical significance.

The comparison of RAGE levels obtained at the first and second visit, i.e., before and after surgical treatment, is shown in Figure 3. The median RAGE levels were 584.2 ng/mL before surgical treatment and increased slightly to 599.7 ng/mL after treatment. However, those results were not statistically significant (*p* = 0.17, Figure 3).

**Figure 4:**
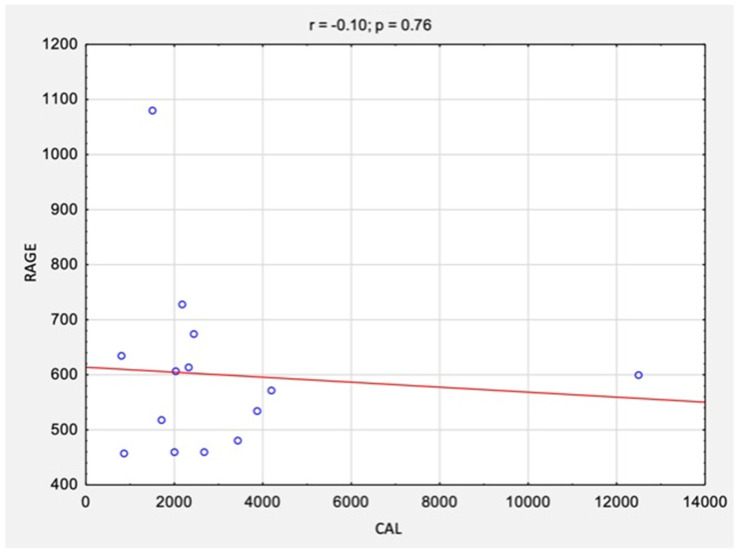
The correlations between the results of CAL levels and RAGE/AGER levels before surgical treatment. *Abbreviations:* r, Spearman’s correlation coefficient; n, number of participants; *p*, level of statistical significance; CAL, calprotectin; RAGE, receptor for advanced glycation end products.

**Figure 5:**
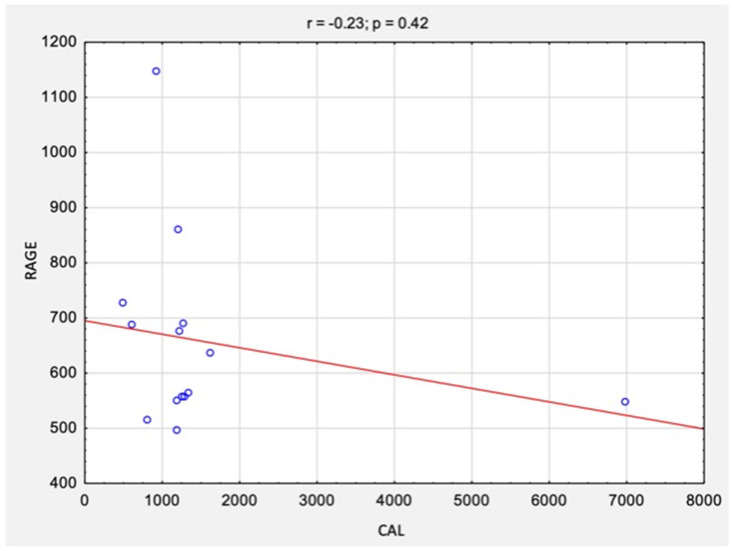
The correlations between the results of CAL and RAGE levels after surgical treatment. *Abbreviations:* r, Spearman’s correlation coefficient; n, number of participants; *p*, level of statistical significance; CAL, calprotectin; RAGE, receptor for advanced glycation end products.

The correlation analysis between the results of CAL levels and RAGE levels before and after surgical treatment is summarized in Table 2 and Figure 4 and Figure 5. There were no statistically significant correlations between the studied variables, neither before surgery (rs = −0.10, *p* = 0.76) nor after surgery (rs = −0.23, *p* = 0.42).

**Figure 6:**
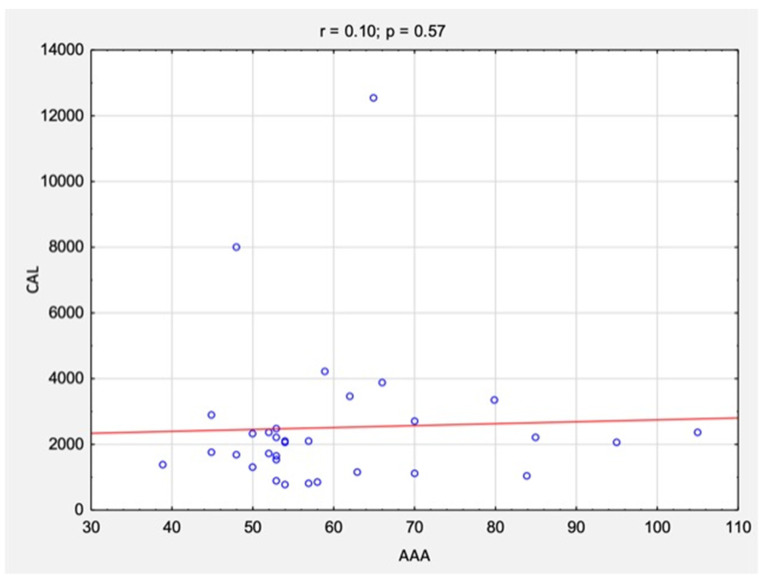
The correlations between the results of CAL levels and AAA size before surgical treatment. *Abbreviations:* r, Spearman’s correlation coefficient; n, number of participants; *p*, level of statistical significance; CAL, calprotectin; AAA, abdominal aortic aneurysm.

**Figure 7:**
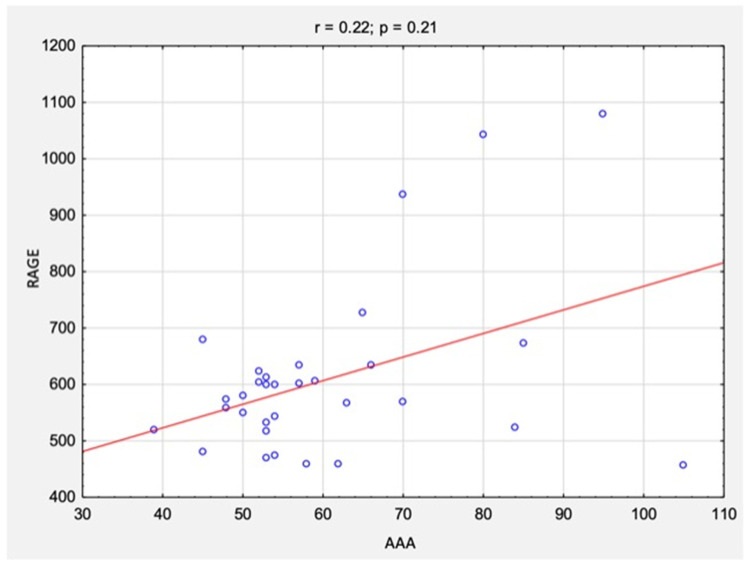
The correlations between the results of RAGE levels and AAA size before surgical treatment. *Abbreviations:* r, Spearman’s correlation coefficient; n, number of participants; *p*, level of statistical significance; RAGE, receptor for advanced glycation end products; AAA, abdominal aortic aneurysm.

The correlation analysis between the results of CAL and RAGE levels and AAA size is summarized in Table 3 and Figure 6 and Figure 7. There were no statistically significant correlations between the studied variables for either CAL levels (rs = 0.10, *p* = 0.57) or RAGE levels (rs = 0.22, *p* = 0.21).

## 4. Discussion

Recent preclinical studies reported a significant correlation between elevated CAL levels and the onset of AAA in humans [4]. Individual human studies concerned RAGE or CAL levels [3,17]. According to previous studies, RAGE and CAL play significant roles in cardiovascular disease and thoracic aortic aneurysms [11,13].

So far, there are no studies, except our previous study [18], which focused on the relationship between elevated CAL and RAGE levels in patients with AAA. The markers mentioned above have not been studied in detail for correlation with AAA diameter so far; hence, there is a limited number of studies on this subject, making it difficult to discuss. In their systematic review, Nana et al. [19] highlighted that circulating serum biomarkers may have applicability in individualized patient surveillance, especially in cases of aggressive AAA growth. There is an urgent need for further studies to obtain reliable data that provide evidence for specific serum biomarkers, potentially informing an individualized surveillance strategy for patients with increased AAA growth.

Interesting results have been presented by Hoshino et al. [20], who assessed plasma calprotectin and other validated biomarkers of subclinical myocardial inflammation (MI) or fibrosis. They considered galectin-3 (Gal-3), growth differentiation factor-15 (GDF-15), soluble ST2 (sST2), and serum procollagen type 1C-terminal propeptide (P1CP). The study group was healthy young adults with a remote history of Kawasaki disease (KD) stratified by coronary artery aneurysm (CAA) status and history of remote MI. It was observed that calprotectin, Gal-3, and GDF-15 levels were significantly higher in subjects with persistent CAA than in subjects with remodeled CAA.

According to preclinical animal studies, no statistically significant correlation exists between elevated levels of CAL and RAGE with AAA size [4]. Nevertheless, these subject needs further methodologically well-designed clinical studies. Initial reports suggest that CAL appears to be a significantly more sensitive marker than RAGE. In our study, CAL showed a statistically significant increase in patients with AAA. Additionally, there was a nearly twofold decrease in CAL levels after AAA repair surgery compared to CAL levels before surgery. Such a noticeable reduction in CAL levels after surgery with removal of a significant area of the AAA wall and removal of an intraluminal thrombus covering 30–50% of the AAA lumen leading to normal blood flow without hemodynamic disturbances may be due to a reduction in the pro-inflammatory response. The impact of biomarkers such as CAL and RAGE should be considered during the analysis of aneurysm pathomechanism. This study suggests that CAL may be a biomarker of the occurrence of AAA.

Currently, the determination of fecal CAL is a marker used in the diagnosis of inflammatory bowel disease (IBD), particularly Crohn’s disease [21,22], monitoring of recovery status for mucosal healing [23,24], and prognosis of IBD exacerbations [25]. The usefulness of CAL determination as a mucosal marker of inflammation has been proved by several studies, systematic reviews, and meta-analyses [26,27]. Furthermore, the fecal CAL levels correlate with neutrophil infiltration intensity in the intestinal mucosa and the severity of inflammation [28,29].

### Study Limitations

This study has some potential methodological and clinical limitations. This clinical and monocentric trial has preliminary character, and further well-designed studies should include multicentric protocol with a larger population of patients with AAA. In this study, we enrolled only one study group, and there were no controls, e.g., healthy volunteers with no AAA diagnosis. Future studies should also consider collecting other morphological parameters and evaluating simple blood counts with neutrophils. Additionally, it would be interesting to provide additional analyses, such as the relationship between CAL or RAGE and endoleaks after repair surgery using traditional OAR or the relationship between CAL levels and fluid dynamics in AAA, as well as including a more extended outcome observation (even a four-year follow-up).

## 5. Conclusions

There were no differences in RAGE levels before and after AAA excision. CAL and RAGE levels were not correlated with AAA size either. However, CAL levels appear to be a significant marker in patients treated surgically for AAA. There was a statistically significant increase in CAL levels in patients with AAA and a nearly twofold decrease in CAL levels in patients after three months following AAA surgery.

## Figures and Tables

**Table 1:** Demographic and clinical characteristics of study participants (n = 32).

Characteristic		Study Group (n = 32)
**Quantitative Variables**		**M ± SD (min–max)**
Age [years]		70.3 ± 7.3 (53–88)
Height [cm]		175 ± 8 (160–190)
Body weight [kg]		91 ± 9.5 (62–120)
BMI [kg/m^2^]		30.5 ± 5 (23–38)
AAA diameter [mm]		60.4 ± 15.0 (39–105)
**Qualitative variables**		**n [%]**
Sex (male)		30 (93.7%)
Hypertension		28 (87.5%)
Type 2 diabetes		5 (14.0%)
Heart disease		9 (28.1%)
AAA size	1a—small	5 (14.0%)
	1b—medium	16 (50.0%)
	1c—large	11 (36.0%)

*Abbreviations:* n, number of participants; M, men; SD, standard deviation; min, minimum value; max, maximum value; AAA, abdominal aortic aneurysm.

**Table 2:** The correlations between the results of CAL levels and RAGE/AGER levels before and after surgical treatment.

Study Group		
	rs	*p*-Value
CAL and RAGE before surgery	−0.10	0.76
CAL and RAGE after surgery	−0.23	0.42

*Abbreviations:* rs, Spearman’s correlation coefficient; *p*, level of statistical significance; CAL, calprotectin; RAGE, receptor for advanced glycation end products.

**Table 3:** The correlations between the results of CAL and RAGE levels and AAA size before surgical treatment.

Study Group		
	rs	*p*-Value
CAL and AAA before surgery	0.10	0.57
RAGE and AAA before surgery	0.22	0.21

*Abbreviations:* rs, Spearman’s correlation coefficient; *p*, level of statistical significance; CAL, calprotectin; RAGE, receptor for advanced glycation end products; AAA, abdominal aortic aneurysm.

## Data Availability

The data presented in this study are available on request from the principal author (W.H.).
